# A Sharp Phase Transition Polymer for High Spatial Selectivity in Microtransfer Printing

**DOI:** 10.1021/acsapm.5c02952

**Published:** 2025-11-19

**Authors:** Jingyang Zhang, Lizhou Yang, Qinhua Guo, Chenchen Zhang, Dong Lu, Yunda Wang

**Affiliations:** † Smart Manufacturing Thrust, 567841 The Hong Kong University of Science and Technology (Guangzhou), Guangzhou 511400, China; ‡ Lab of Future Technology, The Hong Kong University of Science and Technology (Guangzhou), Guangzhou 511400, China; § Department of Mechanical and Aerospace Engineering, The Hong Kong University of Science and Technology, Kowloon, Hong Kong SAR 999077, China

**Keywords:** sharp phase transition polymer, spatial resolution, adhesion modulation, microtransfer printing, flexible electronics

## Abstract

Microtransfer printing (MTP) is an advanced processing technology for flexible electronics manufacturing and heterogeneous integration. As device integration continues to increase, optimizing spatial utilization has become a significant challenge in MTP. In this work, we propose using a reversible, sharp phase transition polymer (SPTP) to enhance the spatial resolution of MTP. In situ characterizations reveal that the SPTP exhibits a pronounced modulus change within a narrow temperature window, corresponding to a distinct phase boundary. This sharp transition enables precise thermal control of adhesion. Dynamic adhesion force measurements further confirm the material’s large adhesion switchability. Leveraging these properties, the SPTP stamp achieves high-resolution selective transfer with a chip spacing of 10 μm. These unique properties of the SPTP provide the material basis for achieving high spatial resolution selective transfer.

## Graphical abstract

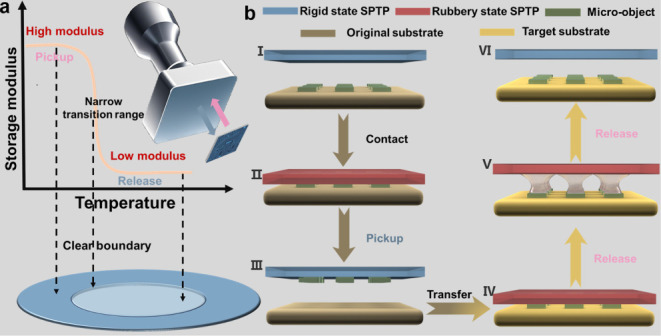

## Introduction

Microtransfer printing (MTP) has emerged as a powerful micro/nanomanufacturing technique, enabling the high-precision transfer of diverse micro- and nanoscale materials from a donor to a receiver substrate.^1^ The compatibility with a broad range of materials, including metals, inorganics, organics, and colloidal materials, as well as its applicability to diverse substrate types such as flexible polymers, glass, ceramics, and metals, has positioned MTP as a key technology for heterogeneous integration in advanced electronics and cellular microstructures.^2–6^ This capability supports numerous applications, including wearable electronics, micro-LED displays, and RF sensors, driving advancements in next-generation electronic devices.^7–10^ Despite these advances, conventional MTP strategies face notable limitations.^11^ The performance of transfer printing mainly depends on the material properties and structure of the stamp, which motivates researchers to focus on the design and optimization of stamps.^12–14^

Recent studies in MTP have mainly focused on improving adhesion switchability to ensure reliable pickup and release during transfer.^13,15^ For instance, phase-change polymers have been used to enable reversible adhesion through melting and crystallization processes, allowing the transfer of objects across various shapes and sizes with minimal loss.^16,17^ Laser-assisted heating has also been applied to significantly increase the adhesion strength of shape memory polymer stamps, achieving switchability ratios over 1000 and enabling precise chip-level transfer.^18–21^ In addition, surface microstructures inspired by biological adhesives have been introduced to control contact area and fine-tune adhesion during different transfer stages.^22^ These approaches have greatly advanced adhesion control by optimizing materials, applying external stimuli, and designing surface features.^23^

However, most of these efforts focus on adhesion control, while relatively little attention has been paid to spatial selectivity. In practical applications, multiple micro-objects are often closely placed on the donor substrate. The ability to transfer a target object without affecting adjacent structures, known as spatial selectivity, is crucial for improving material utilization.^24–26^ Without this capability, unintended pickup or damage may occur, leading to material waste and lower process efficiency.

Stiffness-variable polymers (SVP) represent a class of advanced polymer materials that exhibit significant modulus variations in response to specific environmental stimuli.^27^ Moreover, the reversible and rapid switching of these modulus variations is typically characterized by high repeatability and reliability.^28^ According to the differences in triggering mechanisms and molecular structures, SVPs can be classified into two categories: SVPs based on molecular switches and SVPs based on phase transitions.^29^ In our previous research, we identified an SVP material with rapid phase transition characteristics, capable of undergoing a significant modulus transformation from the rigid state to the rubbery state within a narrow temperature range and possessing a clear boundary.^30,31^ The well-defined and clear boundary between the active and nonactive materials is essential for compatibility with small micro-objects and fine-pitch donors in high spatial resolution selectivity MTP (Figure 1a).

**Figure 1:**
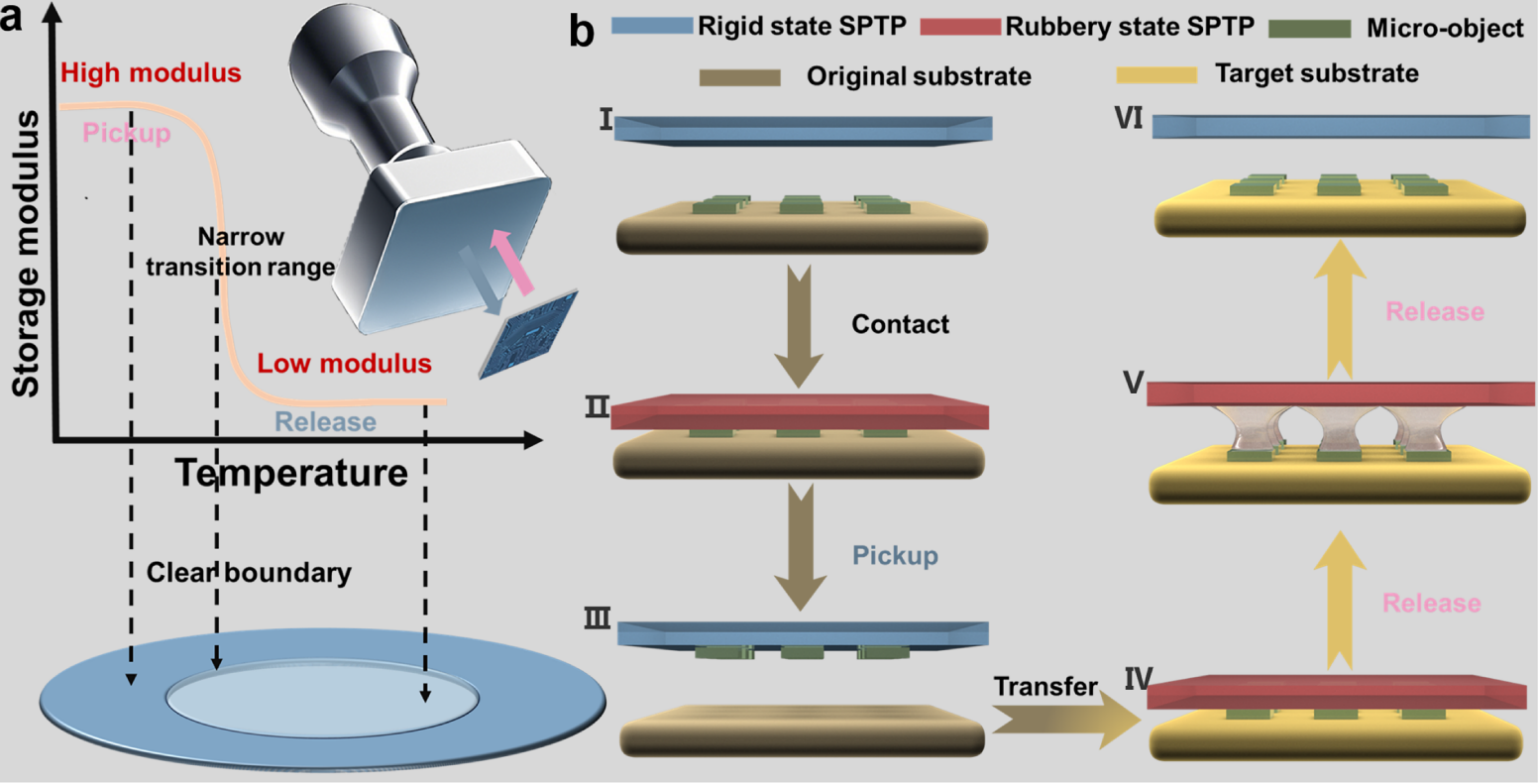
Schematic illustration of the microtransfer printing principle and process of the SPTP stamp. (**a)** The transfer printing mechanism of the SPTP stamp relies on the temperature-modulated storage modulus. (**b)** Schematic illustration of the pickup and printing process of the SPTP stamp. (**I–III** Pickup process) **I** The SPTP stamp approaches the micro-objects on the original substrate. **II** The stamp is heated to enter its rubbery state and is pressed onto the micro-objects, achieving conformal contact due to its low modulus. **III** The stamp is returned to a rigid state with a high modulus, securing the micro-objects firmly and reliably picking up the micro-objects. **(IV–VI** Printing process) **IV** The stamp with micro-objects is moved to the target substrate. **V** The stamp is reheated and returned to its rubbery state with a low modulus and detaches from the micro-objects. **VI** The stamp is fully removed, leaving the micro-object transfer printed on the target substrate.

In this work, we identify and systematically characterize this sharp phase transition polymer (SPTP)^30^ as a promising candidate for high spatial resolution transfer printing. Through the differential scanning calorimetry (DSC) test, it has been proven that a narrow transition range, corresponding to it, possesses the ability for a rapid phase transition. In situ X-ray diffraction (XRD) analysis further reveals that this behavior results from the high-density grafting of long alkyl side chains, which can achieve rapid crystallization and melting transformation within a narrow temperature range. Additionally, in situ atomic force microscopy (AFM) results further show that the SPTP maintains stable viscoelastomer properties across a wide temperature range after the phase transformation, which enables the precise regulation of the dynamic adhesion force of SPTP stamp-based MTP. Adhesion tests using a flat-ended probe demonstrate a 192-fold change in adhesion force, indicating excellent switchability. In transfer printing demonstrations, spatially selective transfer was successfully demonstrated with a 10 μm spacing between the donor chips.

## Results

### Principle and Process of the SPTP Stamp in Microtransfer Printing

The adhesion mechanism of the SPTP stamp involves initial contact in the rubbery state, followed by a transition to the glassy state, which achieves a shape-locking effect that significantly enhances adhesion strength.^33,34^ This process is governed by the temperature-dependent change in the storage modulus of the SPTP, which modifies its viscoelastic properties and enables a reversible transition between strong and weak interfacial adhesion, thereby facilitating pickup and release.^35^ Figure 1b illustrates the principle and process of microtransfer printing using the SPTP stamp. The adhesion modulation process in MTP can be explained using competing fracture theory,^32^ where separation occurs at the interface with the lower critical energy release rate, the minimum energy required to detach an object from a surface. In this process, the detachment happens at either the SPTP/micro-object (P/O) or the micro-object/substrate (O/S) interface. Initially, the SPTP is heated to a rubbery state and made a conformal interface contact with the micro-objects on its original substrate under appropriate preloading. The SPTP stamp is then naturally cooled to become rigid with a high storage modulus, increasing the critical energy release rate between the SPTP and the micro-objects $\left(G_{\mathrm{crit}}^{P/O}\right)$ , which is higher than that between the micro-objects and their original substrate $\left(G_{\mathrm{crit}}^{O/S}\right)$ , enabling strong adhesion for pickup. The SPTP, with the micro-objects attached, is then moved to the target substrate. To release the micro-objects, the SPTP is reheated to its rubbery state with a low modulus, reducing $G_{\mathrm{crit}}^{P/O}$ and making it speed-dependent.^36^ By adjusting the separation speed, $G_{\mathrm{crit}}^{P/O}$ can be kept lower than $G_{\mathrm{crit}}^{O/S}$ , allowing controlled release of the micro-objects onto the target substrate.^37^

### Thermal-Triggered Stiffness Transformation and Phase Transition of SPTP

The SPTP was successfully fabricated through a rapid UV curing process, with stearyl acrylate (SA) and urethane diacrylate (UDA) as the primary raw materials, and trimethylolpropane triacrylate (TMP-TA) serving as the cross-linking agent (Figures S1, S2,and Figure 2a).^30^ By optimizing the ratio of SA and UDA, we obtained the SPTP sample with the maximum modulus transformation. The SPTP used in the subsequent experiments was synthesized in this ratio (80:20) (Figure S3). The long-chain UDA serves as the polymer main chain, improving the elongation at break and enhancing the toughness of the SPTP.^38^ SA, functioning as phase-changing side chains, exhibits a narrow melting temperature range (*T*_*m*_) and is densely grafted onto the main chain in a bottle-brush-like configuration.^39^ TMP-TA, acting as a trifunctional cross-linker, increases the stiffness of the polymer under large strains (Table S1). The stiffness transformation mechanism of SPTP is illustrated in Figure 2b. Below the *T*_*m*_, the long alkyl side chains form crystalline aggregates, rendering the polymer rigid (Figure 2c). Above *T*_*m*_, the side chains adopt an amorphous structure, leading the polymer to transition into a soft and flexible state (Figure 2d). As shown in Figure 2e, the SPTP film exhibits a rigid state at 25 °C, appearing opaque and demonstrating self-supporting properties. At 50 °C, the SPTP film transitions into a rubbery state, becoming transparent. These two states can be reversibly interconverted in response to a temperature change.

**Figure 2:**
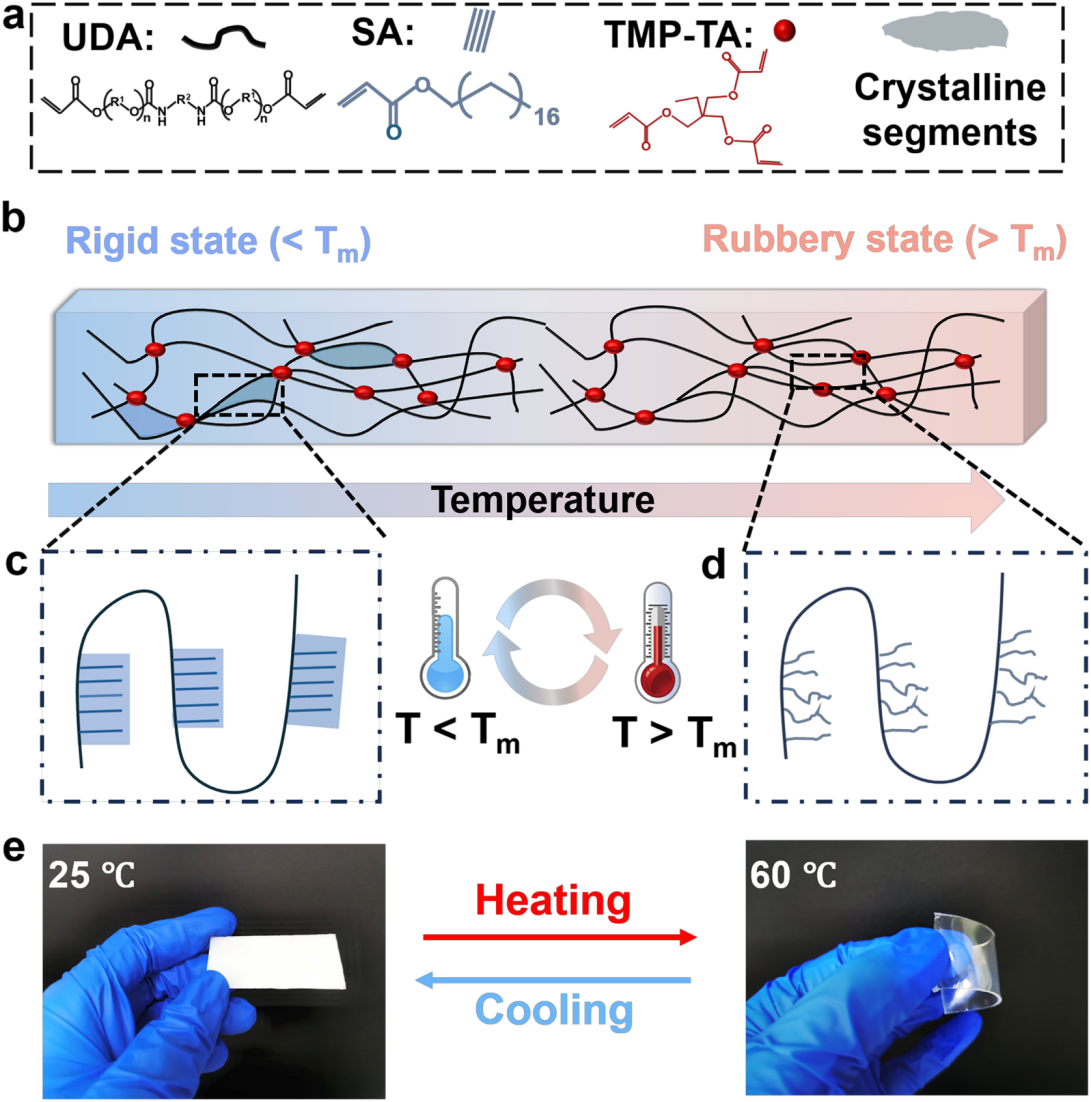
Synthesis of SPTP and the triggering mechanism of stiffness transformation. (**a)** Chemical structure of the primary raw materials. **(b-d)** Schematic illustration of the triggering mechanism for the stiffness transformation of SPTP. (**e)** Photographs of the SPTP film in the rigid state at 25 °C (opaque and hard) and in the rubbery state at 50 °C (transparent and soft). (The film measures approximately 6 cm in length, 2.5 cm in width, and 1 mm in thickness.)

### Thermodynamic and Mechanical Characterization of the SPTP

To investigate the thermodynamic properties of SPTP, we conducted the DSC test. Figure 3a shows the DSC result of SPTP, tested at a consecutive heating/cooling rate of 5 °C/min. The DSC curves exhibited a distinct melting peak during heating and a recrystallization peak during cooling. During the first heating, the melting temperature, indicated by the black dotted line, was recorded as 43.8 °C. Upon subsequent heating-cooling cycles, the *T*_*m*_ remained highly consistent with the initial heating result, with a variation of less than 1 °C. The stable *T*_*m*_ ensures the repeatable use of the SPTP stamp.^40^ The narrow melting range suggests that SPTP undergoes a sharp phase transition from the crystalline state to the molten state, indicating a narrow phase transition region.

**Figure 3:**
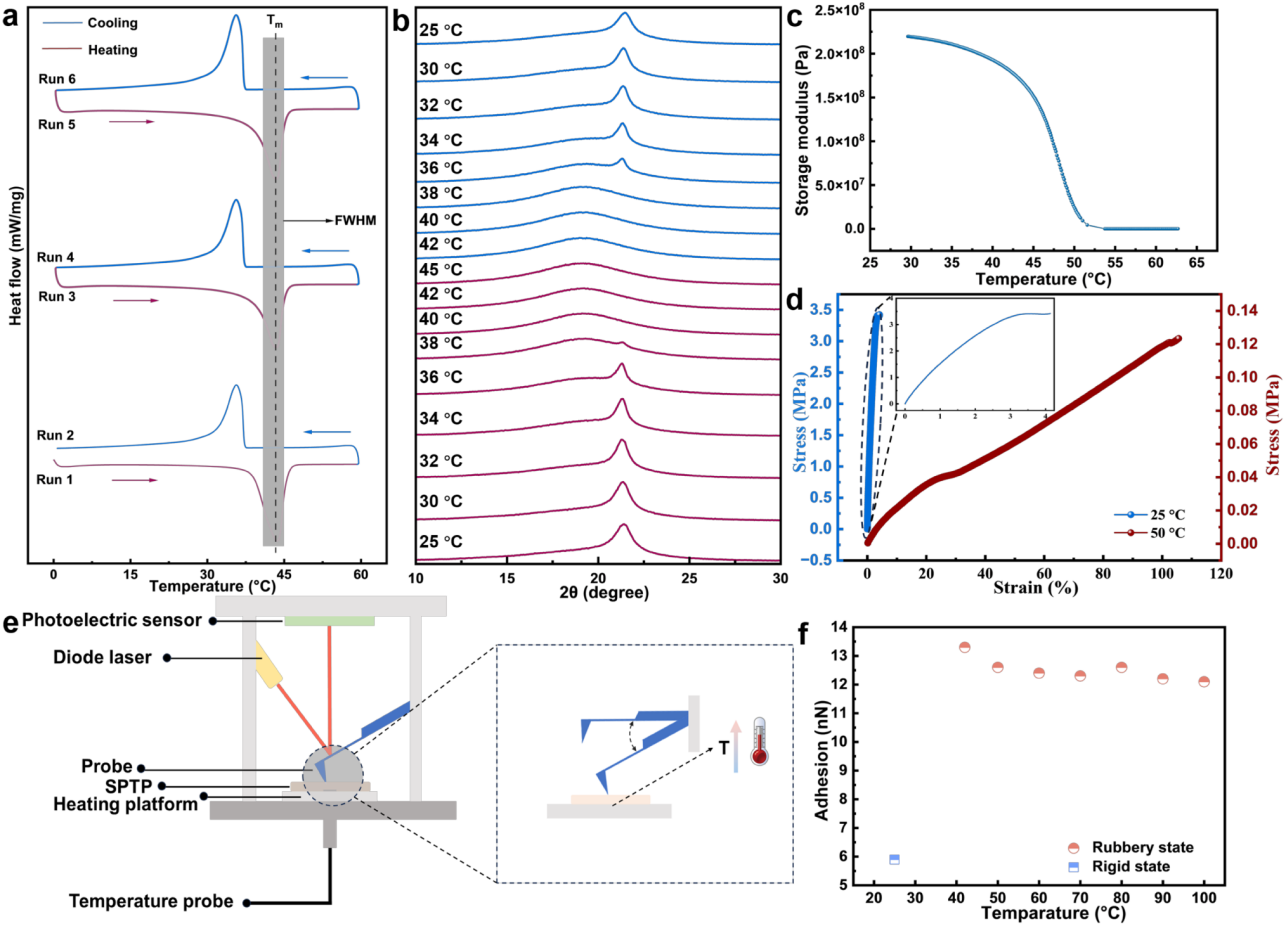
Thermodynamic and mechanical characterization of the SPTP material. (**a)** DSC analysis of SPTP at a rate of 5 °C/min. (**b)** In situ XRD patterns showing structural changes across varying temperatures (25–45–25 °C). (**c)** DMA results show storage modulus as a function of temperature (ramping rate: 2 °C/min). (**d)** Stress–strain curves obtained at 50 and 25 °C at a stretching rate of 3.33 mm/s. (**e)** Schematic illustration of the in situ AFM mechanism and operation modes. (**f)** In situ AFM force data collected from the PeakForce tapping mode across varying temperatures (20–100 °C).

In order to explore the source of the sharp phase transition property, we conducted an in situ XRD test. As shown in Figure 3b, at 25 °C, a sharp single peak, belonging to SA of the α-phase, is observed at 21.4 °C. At this stage, the long alkyl chains of SA are densely packed and aligned in a highly parallel configuration, which enhances the mechanical strength of the SPTP.^41^ Within the temperature range of 36–40 °C, the crystallization peak of SA gradually diminishes until it becomes undetectable, indicating the transition from the crystalline phase to the molten state of SA. This intuitively reflects the sharp phase transition process of SPTP, and the melting transition range is around 4 °C. As the temperature continues to increase until 45 °C, the long alkyl chains of SA are gradually arranged in a disorderly manner.^42^ Upon cooling back to 36 °C, the crystallization peak of SA reappears, corresponding to the recrystallization process. The in situ XRD provides a real-time monitoring method that visually reflects the phase transition process of SPTP and the corresponding temperature window. It was directly proved that the SA component played a role similar to a “switch” in the phase transition process of SPTP, dominating the phase transition of the overall polymer network. This clarified the source of the rapid phase transition property of SPTP and provides guidance for the development of materials with a rapid phase transition property. Figure 3c shows the DMA result of the SPTP, conducted at a temperature ramping rate of 2 °C/min and a frequency of 1 Hz across a temperature range of 30–60 °C. At room temperature, the storage modulus is measured at 219.6 MPa, attributed to crystalline aggregates of the SA moiety acting as hard segments. As the temperature increases, these aggregates melt, leading to a sharp modulus decrease to 145.8 kPa. This transition, involving a 1506-fold decrease in storage modulus, occurs within a narrow temperature range of approximately 4 °C. This rapid, temperature-dependent transition enables precise spatial control of adhesion, allowing selective transfer from densely packed arrays without affecting adjacent devices. Both DMA and XRD results confirm the sharp transition of SPTP, which is highly advantageous for high spatial resolution selective MTP. The tensile stress-strain responses of the SPTP samples were measured in both the rubbery and the rigid states. At 50 °C, in the rubbery state, the material exhibits a tensile strength of 0.12 MPa and an elongation at break of 104 %, suggesting good flexibility (red curve in Figure 3d). At 25 °C, in the rigid state, the material shows a higher tensile strength of 6.43 MPa and a reduced elongation at break of 4.13% (blue curve in Figure 3d). The stress-strain test results suggest that the SPTP’s properties allow it to adapt to micro-objects in the rubbery state for secure contact, while its strength in the rigid state provides reliable support during pickup.^43^ To further evaluate its thermal tolerance, we also conducted thermogravimetric analysis (TGA) to determine the maximum allowable operating temperature of the material. As shown in Figure S4, the SPTP framework exhibits excellent thermal stability from 25 to 178 °C. Gradual decomposition begins around 178 °C, followed by rapid degradation into carbon dioxide and other byproducts between 350 and 435 °C. For MTP applications, the phase transition temperature of the stamp is typically required to remain below 100 °C to prevent potential damage to the transferred materials.

Furthermore, we evaluated the behavior of the surface adhesion of the SPTP material as it varies with temperature. The AFM is recognized as a powerful tool for characterizing the surface adhesion force of materials at the microscale.^44,45^ Using in situ AFM technology, we systematically measured the variation in the surface adhesion of SPTP under continuous heating conditions (Figure 3e). As shown in Figure 3f, it is evident that when the temperature is below *T*_*m*_, SPTP remains in a rigid state, characterized by low internal molecular chain mobility, resulting in an interfacial adhesion measurement of 6 nN.^46^ In contrast, when the temperature is above *T*_*m*_, SPTP undergoes a phase transition to the rubbery state. In this state, the enhanced molecular chain mobility leads to a significant increase in interfacial adhesion, with the measured value reaching 13 nN.^47^ Moreover, once the temperature is above *T*_*m*_, the interfacial adhesion force of SPTP remains stable across a broad temperature range from 40 to 100 °C. This indicates that the material exhibits excellent thermal stability and reliable interfacial adhesion performance. These properties simplify the parameter control required for dynamic adhesion modulation in the MTP process.

### Dynamic Adhesion Modulation Mechanism and Characterization

To explore the adhesion modulation mechanics of the SPTP stamp, including the effect of preload, temperature, and separation speed on performance, we first customized a 90 ° vertical separation test setup (Figures 4a and S5). Adhesion data were captured using a high-precision load cell operating at a sampling rate of 4800 Hz and an accuracy of 0.001 g. The flat surface SPTP stamp (7.5 × 2.5 × 1 mm^3^) was prefabricated on a 1 mm-thick glass substrate, and an aluminum alloy test tip (500 μm diameter) was used for measurements (Figure S6). The details of the two test modes are listed in Figure 4a. Force data were recorded during the approaching, preloading, holding, and separating processes, with the adhesion strength defined as the peak of pull-off force during separation, measured at varying conditions shown in Figure 4b and Figure S7. To balance the heat transfer and dissipation in the open space and to determine the actual temperature at which the contact interface undergoes a complete phase transition, we conducted experiments with varying temperature gradients. Contacts were held for 5 min before separation, and separation forces were recorded (Figure 4c). The results show that when the hot plate exceeds 60 °C, the phase transition at the contact interface is fully completed, as indicated by stable adhesive forces during separation. Next, the hot plate was set to 60 °C and maintained for 5 min to ensure sufficient interfacial heat transfer prior to investigating the mechanism of adhesion force regulation through separate speed control. To further evaluate the stamp’s durability, we conducted continuous adhesion-separation tests. The heating source was maintained at 60 °C, the preload was 250 kPa, and the test tip remained in contact with the stamp surface for 5 min before being separated at a speed of 1 μm/s. As shown in Figure S8, during 25 consecutive preload and release cycles, the adhesive force remained relatively stable without a significant decrease, demonstrating the material’s good durability and reliability for long-term use.

**Figure 4:**
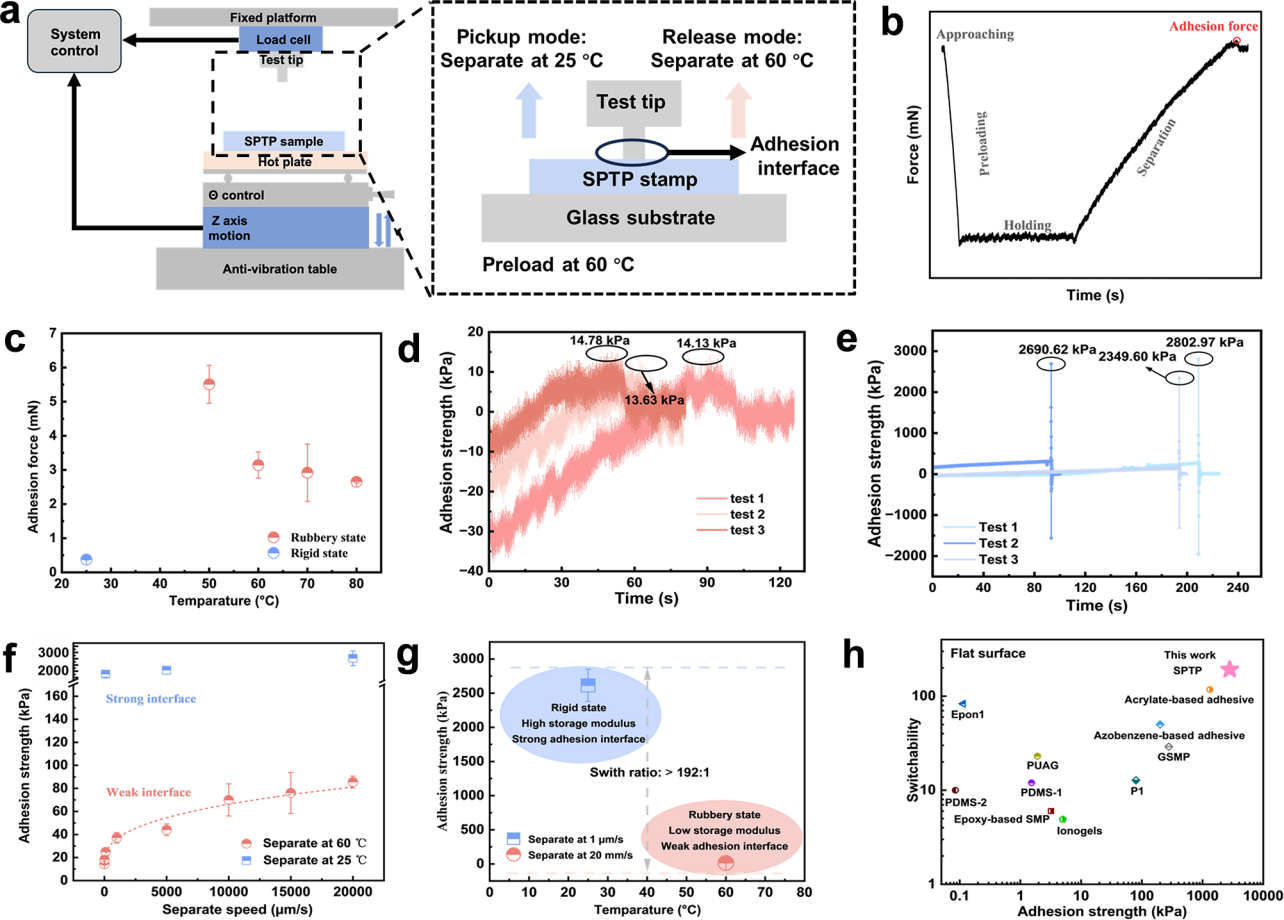
Characterization and modulation of interface adhesion. (**a)** Schematic diagram of the customized adhesion strength measurement system. Enlarged image: Details of the adhesion test conditions for release and pickup modes using the SPTP stamp. (**b)** A representative force-time graph of the adhesion test. (**c)** Interfacial adhesion strength at different set temperatures of the hot plate. (**d)** Adhesion strength of the SPTP stamp in release mode (three repeated tests). (**e)** Adhesion strength of the SPTP stamp in pickup mode. (The three highest values from over 10 repeated tests were selected for analysis.) (**f)** Interfacial adhesion strength at different temperatures and separate speeds. (**g)** Interface adhesion switch ratio of the SPTP stamp. (**h)** Plot for adhesion strength and switchability comparison (details in Table S2).

To characterize the adhesion-switching properties of the SPTP stamp, we measured the adhesion force (F) in pickup and release modes. The adhesion strength (σ = F/A) was calculated using F and a 500 μm-diameter circular contact area (A = π×250^2^ μm^2^). Figure S9 illustrates the adhesion force test process, and a 250 kPa preload (the preload depth is about 50 μm, as shown in Figure S10) was applied to bring the SPTP stamp and the test tip interface into full contact. To obtain more accurate and reliable adhesion force data, different sampling strategies were adopted for the release and pickup modes. For the release mode, as shown in Figure 4d, the separation process is relatively slow, allowing for a sufficient sampling frequency to capture the adhesion force accurately. Therefore, three repeated tests were conducted, and their average was taken as the final result. The pickup mode involves instant separation, as shown in Figure 4e, which often leads to undersampling and difficulty in capturing the true peak force. As a result, the measured average tends to underestimate the actual adhesion strength. To address this, over ten tests were performed for each condition, and the three highest values were averaged to better approximate the actual peak adhesion.

Figure 4f shows the relationship between the adhesion strength and the separation speed of the SPTP stamp/aluminum alloy test tip (P/A) interface. In pickup mode, the rigid SPTP and test tip are both regarded as an elastomer. The (Rigid P/A) interface is a strong adhesion interface and is considered as a material property that is independent of the separation speed. The average adhesion strength is 2108.23, 2230.74, and 2614.40 kPa at the separation speeds of 100 μm/s, 5000 μm/s, and 20000 μm/s, respectively. The adhesion strength increases slightly with increasing speed, mainly because the hard SPTP, despite its rigid state, is not an ideal elastic material. However, overall, when tested at different orders of separation speeds, the adhesion strength remains the same order of magnitude. Recent studies have indicated that transfer printing using kinetically switchable adhesion to an elastomeric stamp can be modeled as the competing fracture of two interfaces.^27^ In release mode, the SPTP is in its rubbery state, acting as a viscoelastomer with deformation ability, and the adhesion of the interface (Rubbery P/A) is sensitive to the separation rate. The pink data points in Figure 4f correspond to the release process, where increasing the separation speed increases the interfacial adhesion. This relationship between interfacial adhesion and separation speed can be mathematically represented by the equation.^48^

1 $$\sigma_{\mathrm{crit}}^{\left(P/A\right)}\left(v\right)=12.31\left[1+{\left(\frac{v}{152.82}\right)}^{0.35}\right]$$

The fitting results are represented by the dashed lines in Figure 4f, showing a good fit when the R^2^ value is 0.97727. The ratio of maximum to minimum adhesion is referred to as the “adhesion strength ratio” of the transfer printing process. This ratio characterizes the switchability of a stamp in MTP, where a larger adhesion strength ratio generally indicates enhanced transfer reliability. When the separation speed is slow at 1 μm/s in release mode, the adhesion strength is about 14.6 kPa. The highest adhesion strength at 20 mm/s in pickup mode we tested is about 2802.97 kPa. As illustrated in Figure 4g, the SPTP demonstrates an impressive adhesion strength ratio of approximately 192:1 when tested using a flat-surfaced SPTP stamp against a flat test tip, ensuring a nonembedded contact interface. To ensure a fair and consistent comparison, the results were compared with those of other switchable polymer stamps featuring flat contact interfaces and adhesion primarily governed by the modulus change, as shown in Figure 4h. The SPTP stands out for its excellent adhesion switchability, and strong overall adhesive strength.^49^

### Demonstration of High Spatial Resolution Selective MTP Enabled by SPTP Stamp

The sharp phase transition property of the SPTP stamp endows it with a tunable working area and a well-defined boundary, which is essential for achieving high spatial selectivity in transfer, as illustrated in Figure 5a. To validate this, we systematically investigated the working area of the stamp by modulating the applied power. The SPTP stamp used in this work has a thickness of approximately 28 μm, at which the material remains semitransparent to visible light. This partial translucency facilitates optical alignment between the stamp and donor substrate during the transfer process. The results are shown in Figure 5b: as the applied heating power—supplied through the stamp substrate by a small ceramic heater—gradually increases from 1.27 to 1.60 W, the active area continuously expands, with the diameter increasing from 3.32 mm to 5.39 mm. Due to the significant change in the transparency of the SPTP before and after phase transition, we can observe a clear boundary, providing direct visual evidence to distinguish the working and nonworking areas. To evaluate the practical effectiveness of the SPTP stamp in high-resolution spatially selective MTP, we conducted experiments using a 3 × 3 densely packed photoresist (PR) chiplet array with only 10 μm spacing (PR chiplet size: ∼ 50 μm × 50 μm × 15 μm). The selective picking and releasing process is shown in the schematic diagram in Figure S11. The localized heating elements used in this demonstration were implemented using microfabricated thin-film heaters, which enable precise temperature control at the microscale. As shown in Figure 5c, after the merge processing of the chiplet microscope images before and after transfer through linear transformation with the assistance of Fiji software, we successfully transferred only the central chiplet without disturbing adjacent units. This result clearly demonstrates the high spatial resolution achievable with the SPTP film and highlights the practical utility of its sharp thermal phase transition in enabling “point-to-point” accuracy MTP with space utilization limitations.

**Figure 5:**
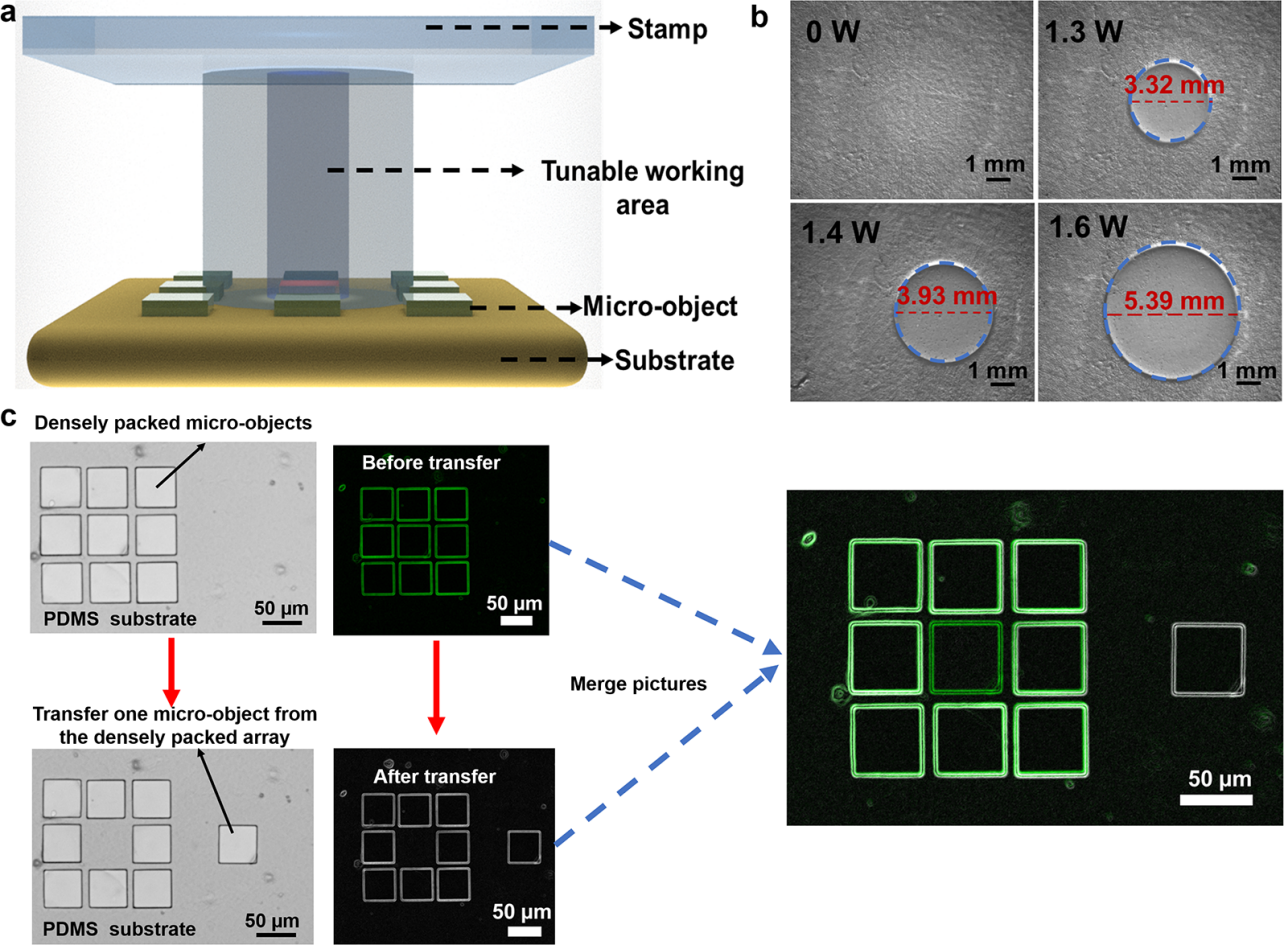
Demonstration of high spatial resolution selective MTP based on the sharp phase transition property of the SPTP stamp. (**a**) Schematic diagram of the tunable work area of the SPTP stamp set by setting the power. (**b**) The microscopic images of the SPTP film surface with different heating powers. (**c**) The microscopic images of the high-resolution selective MTP and the overlay results of the PR structure before and after transfer (photoresist structure size: 50 × 50 × 15 μm, pixel spacing: 10 μm, 3 × 3 closely packed array).

### Demonstration of Transfer Printing Different Micro-objects

To evaluate the adhesion switchability of the SPTP stamp, we conducted experiments with different objects and substrates to demonstrate the microtransfer printing process. These objects were transferred using a 1 mm-thick SPTP stamp fabricated on a 1 mm-thick glass substrate (Figure S5a), and the process was carefully recorded by an optical microscope. Figure 6a shows the transfer of a 50 × 50 × 15 μm PI square ring array from a PDMS substrate to another PDMS substrate. Both the original substrate and the target substrate are PDMS films with the same surface conditions. This successful transfer demonstrates the SPTP material’s efficient adhesion switchability, while also indicating the material’s potential use to effectively repopulate the micro-objects on the same substrate. It is noteworthy that the “square ring” structure is prone to deformation or tearing. The successful transfer also indicates that the low modulus of the SPTP stamp is suitable and has good control over the mechanical stress distribution of thin-film structures, which is applicable for handling devices with hollow or fragile structures. The PI substrate is a commonly used material in flexible electronics. Transferring hard materials such as Si chiplets to PI substrates is a key step in achieving flexible integrated circuits, wearable devices, and flexible sensors, etc. Therefore, we experimented with transferring a 6 × 6 silicon cube array from a PDMS substrate to the PI substrate. As shown in Figure 6b, all the 36 micro-objects were transferred at one time, indicating that the interface interaction between the SPTP stamp and PDMS, PI substrate is uniformly controlled. This is very crucial for designing programmable transfer printing and achieving a precise release. In addition, the effective transfer of both flexible PI and rigid Si chiplets, demonstrates the compatibility of the adhesion-tunable SPTP stamp with heterogeneous materials and its suitability for the heterogeneous integration of complex microdevices.

**Figure 6:**
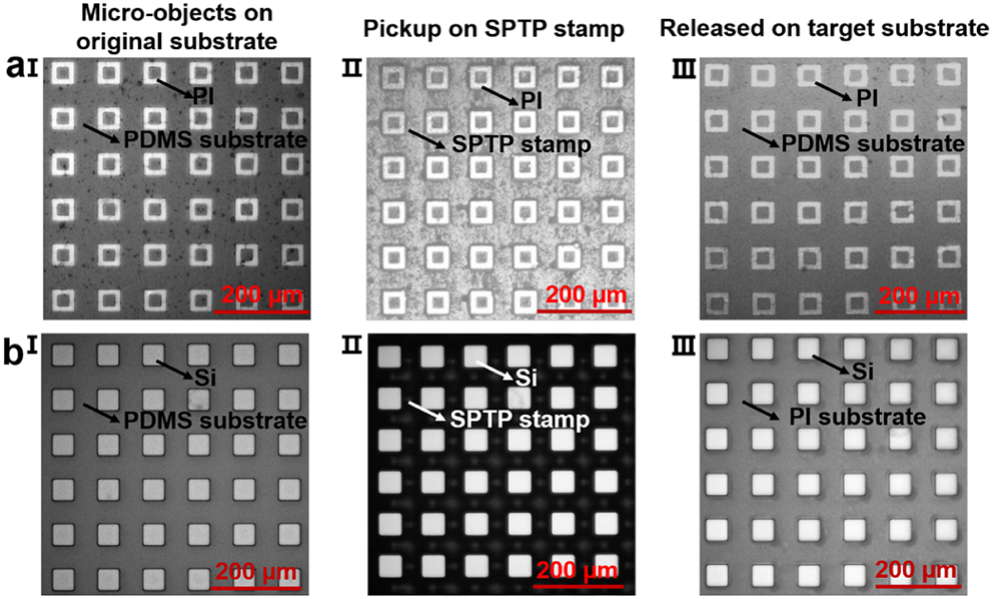
Demonstration of transfer printing different micro-objects. (**a)** Optical microscopy images of the 50 × 50 × 15 μm polyimide (PI) square rings array transfer printing process. I. The 6 × 6 PI array on the original PDMS substrate. II. The PI array was transferred onto the SPTP stamp surface. III. The PI array was released onto another target PDMS substrate (having the same surface adhesion conditions as the original). (**b)** Optical microscopy images of the 50 × 50 × 75 μm silicon (Si) cube array transfer printing process. I. The 6 × 6 Si array on the original PDMS substrate. II. The Si array was transferred onto the SPTP stamp surface. III. The Si array was released onto the PI target substrate.

## Conclusion

In summary, we have proposed a reversible sharp phase transition polymer as a model material for high-resolution selective transfer. This polymer is synthesized using a simple UV curing method. Comprehensive material characterization results indicate that SPTP exhibits a narrow melting range, resulting in a well-defined phase boundary. The dynamic adhesion tests reveal that it has a large and variable adhesion strength ratio of 192 times. These properties demonstrate the strong potential of SPTP for applications in high-resolution selective microtransfer printing.

## Methods

### Materials

Urethane diacrylate (UDA) was obtained from Sartomer Co. and used as received. Stearyl acrylate (SA), trimethylolpropane triacrylate (TMP-TA), 2,2-dimethoxy-2-phenyl-acetophenone (DMPA), and benzophenone (BP) were purchased from Sigma-Aldrich and used without further purification. The adhesion promoter (3 M 94 Primer) was acquired from 3M, USA. Silicone gel film was sourced from Gel-Pak, USA, and polydimethylsiloxane (PDMS, SYLGARD 184) was obtained from Dow Corning, USA. AZ5214E photoresist was provided by AZ Electronic Materials, USA. Polyimide film (SMW-610) was obtained from Changzhou Runchuan Plastic Material Co., Ltd., China. A single-axis load cell (LRM200, 100 g range) was purchased from Futek, USA.

### Fabrication of the SPTP Stamp

Two types of SPTP stamps were prepared. For adhesion characterization, a 1 mm-thick SPTP layer was fabricated by heating the prepolymer solution until transparent and then dispensing it between two heated glass plates separated by a 1 mm tape spacer. The assembly was UV-cured (365 nm, 2000 mW/cm^2^, ambient conditions, 10 rpm rotation) for ∼5 min, then annealed at 80 °C for 2 h. After cooling naturally, the antistick-coated top glass plate was removed to obtain the SPTP stamp. A thinner SPTP layer (∼28 μm) for μTP experiments was similarly fabricated by using a 28-μm Kapton tape spacer.

### Adhesion Force Test

Initially, the stamp was heated and establishing conformal contact under a preload of 250 kPa, kept for 5 min to complete the heat transfer. For the pickup mode test, turn off the hot plate, allowing the sample and interface to naturally cool below *T*_*m*_ before separation. For the release mode test, separation occurred while maintaining the 80 °C hot plate setting. The contact speed is 10 μm/s, and the separation speed is set from 10 to 20 mm/s. Adhesion force was quantified as the peak pull-off force during the separation phase and was recorded under varying temperature, separate speed, and material conditions.

### Microtransfer Printing Process of the Micro-objects Array

The micro-object array with a pitch of 100 μm was prepared by doing photolithography on the original substrate after surface hydrophilic treatment. For the pickup step, the SPTP stamp is heated on a hot plate set to 80 °C for 1 min, allowing it to reach a rubbery state. It is then pressed onto the micro-objects on the original substrate with a force of about 10 N to ensure full contact. After natural cooling to room temperature, the substrate is separated at a speed of about 5 μm/s, picking up the micro-objects onto the stamp surface. For the release step, the stamp carrying the micro-objects is reheated and pressed onto the target substrate, and then the stamp is separated at a lower speed of about 1 μm/s.

### Characterizations

Fourier Transform Infrared Spectroscopy (FTIR) was conducted using a Bruker-Vertex 70 V instrument to analyze SA-UDA mixtures and prepolymer samples cured for different durations, with spectra collected over 2000–500 cm^– 1^. Differential Scanning Calorimetry (DSC) was performed after preannealing samples at 60 °C to eliminate thermal history. Dynamic Mechanical Analysis (DMA) was carried out on a TA Instruments DMA 850 with temperature sweeps from 25 °C to 60 °C at 1 Hz and tensile tests at 25 °C and 50 °C. In situ X-ray diffraction (XRD) measurements were performed using a Malvern PANalytical Empyrean 3.0 in the range of 25 °C to 45 °C, with peak separation analysis done via OriginPro 8.

## Supplementary Material



## Data Availability

The data that support the findings of this study are present in the article and the Supporting Information. Additional data related to this study are available from the corresponding author upon request.

## Notes

Additional experimental details, materials, and methods, including photographs of the experimental setup (PDF)

## References

[1] Park J., Lee Y., Lee H., Ko H. (2020). Transfer Printing of Electronic Functions
on Arbitrary Complex Surfaces. ACS Nano.

[2] Cheng X., Fan Z., Yao S., Jin T., Lv Z., Lan Y., Bo R., Chen Y., Zhang F., Shen Z. (2023). Programming
3d Curved Mesosurfaces Using Microlattice Designs. Science.

[3] Hui Z., Zhang L., Ren G., Sun G., Yu H.-D., Huang W. (2023). Green Flexible Electronics: Natural
Materials, Fabrication, and Applications. Adv.
Mater..

[4] Hu L., Chee P. L., Sugiarto S., Yu Y., Shi C., Yan R., Yao Z., Shi X., Zhi J., Kai D., Yu H.-D. (2023). Hydrogel-Based Flexible
Electronics. Adv. Mater..

[5] Bo R., Xu S., Yang Y., Zhang Y. (2023). Mechanically-Guided
3D Assembly for
Architected Flexible Electronics. Chem. Rev..

[6] Matsuhisa N., Niu S., O’Neill S. J. K., Kang J., Ochiai Y., Katsumata T., Wu H.-C., Ashizawa M., Wang G.-J. N., Zhong D. (2021). High-Frequency and Intrinsically Stretchable
Polymer Diodes. Nature.

[7] Yang Y., Gao W. (2019). Wearable and Flexible
Electronics for Continuous Molecular Monitoring. Chem. Soc. Rev..

[8] Zhou X., Tian P., Sher C.-W., Wu J., Liu H., Liu R., Kuo H.-C. (2020). Growth, Transfer
Printing and Colour Conversion Techniques
Towards Full-Colour Micro-Led Display. Prog.
Quantum Electron..

[9] Choi M. K., Yang J., Kang K., Kim D. C., Choi C., Park C., Kim S. J., Chae S. I., Kim T.-H., Kim J. H. (2015). Wearable Red–Green–Blue Quantum Dot Light-Emitting
Diode Array Using High-Resolution Intaglio Transfer Printing. Nat. Commun..

[10] Jeong J.-W., McCall Jordan J., Shin G., Zhang Y., Al-Hasani R., Kim M., Li S., Sim Joo J., Jang K.-I., Shi Y. (2015). Wireless Optofluidic Systems for Programmable In vivo Pharmacology
and Optogenetics. Cell.

[11] Liu L., Cai Z., Xue S., Huang H., Chen S., Gou S., Zhang Z., Guo Y., Yao Y., Bao W., Zhou P. (2025). A Mass Transfer Technology
for High-Density Two-Dimensional Device
Integration. Nat. Electron..

[12] Shi C., Jiang J., Li C., Chen C., Jian W., Song J. (2024). Precision-Induced Localized
Molten Liquid Metal Stamps for Damage-Free
Transfer Printing of Ultrathin Membranes and 3D Objects. Nat. Commun..

[13] Li Y., Zhang F., Wang S. (2024). Regulatable
Interfacial Adhesion
between Stamp and Ink for Transfer Printing. Interdiscip. Mater..

[14] Luo H., Wang C., Linghu C., Yu K., Wang C., Song J. (2020). Laser-Driven Programmable Non-Contact Transfer Printing of Objects
onto Arbitrary Receivers Via an Active Elastomeric Microstructured
Stamp. Natl. Sci. Rev..

[15] Linghu C., Zhang S., Wang C., Yu K., Li C., Zeng Y., Zhu H., Jin X., You Z., Song J. (2020). Universal Smp Gripper with Massive and Selective Capabilities
for
Multiscaled, Arbitrarily Shaped Objects. Sci.
Adv..

[16] Linghu C., Liu Y., Yang X., Li D., Tan Y. Y., Mohamed
Hafiz M. H. B., Rohani M. F. B., Du Z., Su J., Li Y. (2024). Fibrillar Adhesives with Unprecedented Adhesion Strength,
Switchability and Scalability. Natl. Sci. Rev..

[17] Chen L., Liang H., Liu P., Shu Z., Wang Q., Dong X., Xie J., Feng B., Duan H. (2024). Phase-Change
Stamp with Highly Switchable Adhesion and Stiffness for Damage-Free
Multiscale Transfer Printing. ACS Nano.

[18] Eisenhaure J., Kim S. (2016). Laser-Driven Shape Memory Effect for Transfer Printing Combining
Parallelism with Individual Object Control. Adv. Mater. Technol..

[19] Huang Y., Zheng N., Cheng Z., Chen Y., Lu B., Xie T., Feng X. (2016). Direct Laser
Writing-Based Programmable Transfer Printing
Via Bioinspired Shape Memory Reversible Adhesive. ACS Appl. Mater. Interfaces.

[20] Luo H., Li C., Wang S., Zhang S., Song J. (2024). Switchable Adhesive
Based on Shape Memory Polymer with Micropillars of Different Heights
for Laser-Driven Noncontact Transfer Printing. ACS Appl. Mater. Interfaces.

[21] Li C., Zhang S., Jiang J., Wang S., He S., Song J. (2024). Laser-Induced Adhesives
with Excellent Adhesion Enhancement and Reduction
Capabilities for Transfer Printing of Microchips. Sci. Adv..

[22] Kim S., Wu J., Carlson A., Jin S. H., Kovalsky A., Glass P., Liu Z., Ahmed N., Elgan S. L., Chen W. (2010). Microstructured
Elastomeric Surfaces with Reversible Adhesion and Examples of Their
Use in Deterministic Assembly by Transfer Printing. Proc. Natl. Acad. Sci. U. S. A..

[23] Jian W., Chen Y., Feng X. (2025). 3D Conformal
Curvy Electronics: Design,
Fabrication, and Application. ACS Nano.

[24] Song D., Mahajan A., Secor E. B., Hersam M. C., Francis L. F., Frisbie C. D. (2017). High-Resolution Transfer Printing of Graphene Lines
for Fully Printed, Flexible Electronics. ACS
Nano.

[25] Lee S., So C., Sim K. M., Son C., Chung D. S., Kim S. (2023). Ultrathin,
Flexible, and Reusable Silicon Shadow Masks Manipulated via Transfer
Printing. Adv. Mater. Technol..

[26] Chen Y., Shu Z., Feng Z., Kong L. A., Liu Y., Duan H. (2020). Reliable Patterning,
Transfer Printing and Post-Assembly of Multiscale Adhesion-Free Metallic
Structures for Nanogap Device Applications. Adv. Funct. Mater..

[27] Apsite I., Biswas A., Li Y., Ionov L. (2020). Microfabrication Using
Shape-Transforming Soft Materials. Adv. Funct.
Mater..

[28] Montero
de Espinosa L., Meesorn W., Moatsou D., Weder C. (2017). Bioinspired
Polymer Systems with Stimuli-Responsive Mechanical Properties. Chem. Rev.

[29] Gao M., Meng Y., Shen C., Pei Q. (2022). Stiffness Variable
Polymers Comprising Phase-Changing Side-Chains: Material Syntheses
and Application Explorations. Adv. Mater..

[30] Ren Z., Hu W., Liu C., Li S., Niu X., Pei Q. (2016). Phase-Changing
Bistable Electroactive Polymer Exhibiting Sharp Rigid-to-Rubbery Transition. Macromolecules.

[31] Zhang, J. ; Shu, X. ; Guo, Q. ; Lu, D. ; Wang, Y. A Sharp Phase Transition Shape Memory Polymer for Micro-Transfer Printing. In 2024 IEEE 19th International Conference on Nano/Micro Engineered and Molecular Systems (NEMS)); IEEE, 2024.

[32] Meitl M. A., Zhu Z.-T., Kumar V., Lee K. J., Feng X., Huang Y. Y., Adesida I., Nuzzo R. G., Rogers J. A. (2006). Transfer
Printing by Kinetic Control of Adhesion to an Elastomeric Stamp. Nat. Mater..

[33] Linghu C., Liu Y., Tan Y. Y., Sing J. H. M., Tang Y., Zhou A., Wang X., Li D., Gao H., Hsia K. J. (2023). Overcoming
the Adhesion Paradox and Switchability Conflict on Rough Surfaces
with Shape-Memory Polymers. Proc. Natl. Acad.
Sci. U. S. A..

[34] Linghu C., Yang X., Liu Y., Li D., Gao H., Hsia K. J. (2023). Mechanics of Shape-Locking-Governed R2G Adhesion with
Shape Memory Polymers. J. Mech. Phys. Solids.

[35] Linghu C., Mao W., Jiang H., Gao H., Hsia K. J. (2025). Rubber-to-Glass
Adhesion between a Rigid Sphere and a Shape Memory Polymer Substrate
of Finite Thickness. Int. J. Solids Struct..

[36] Gong L. (2025). Thermal- and
Rate-Regulated Fast Switchable Adhesion within Glass Transition Zone
of an Epoxy Polymer. Langmuir.

[37] Gong L., Wang X. (2021). Thermal-Regulated Adhesion Enhancement and Fast Switching within
the Viscoelastic Glass Transition Zone of a Shape Memory Polymer. Langmuir.

[38] Staszczak M., Nabavian Kalat M., Golasiński K.
M., Urbański L., Takeda K., Matsui R., Pieczyska E. A. (2022). Characterization
of Polyurethane Shape Memory Polymer and Determination of Shape Fixity
and Shape Recovery in Subsequent Thermomechanical Cycles. Polymers.

[39] Liu K., Zhang Y., Cao H., Liu H., Geng Y., Yuan W., Zhou J., Wu Z. L., Shan G., Bao Y. (2020). Programmable Reversible Shape Transformation of Hydrogels
Based on Transient Structural Anisotropy. Adv.
Mater..

[40] Qiu Y., Lu Z., Pei Q. (2018). Refreshable
Tactile Display Based on a Bistable Electroactive
Polymer and a Stretchable Serpentine Joule Heating Electrode. ACS Appl. Mater. Interfaces.

[41] Yao M., Nie J., He Y. (2018). Can Chain-Reaction
Polymerization of Octadecyl Acrylate
Occur in Crystal?. Macromolecules.

[42] Gao M., Wu H., Plamthottam R., Xie Z., Liu Y., Hu J., Wu S., Wu L., He X., Pei Q. (2021). Skin Temperature-Triggered
Debonding-on-Demand Sticker for a Self-Powered Mechanosensitive Communication
System. Matter.

[43] Aguirresarobe R. H., Nevejans S., Reck B., Irusta L., Sardon H., Asua J. M., Ballard N. (2021). Healable and
Self-Healing Polyurethanes
Using Dynamic Chemistry. Prog. Polym. Sci..

[44] Petroli A., Petroli M., Romagnoli M., Geoghegan M. (2022). Determination
of the Rate-Dependent Adhesion of Polydimethylsiloxane Using an Atomic
Force Microscope. Polymer.

[45] Zhai Z., Schmid S. Y., Lin Z., Zhang S., Jiao F. (2024). Unveiling
the Nanoscale Architectures and Dynamics of Protein Assembly with
in Situ Atomic Force Microscopy. Aggregate.

[46] Liang R., Yu H., Wang L., Amin B. U., Wang N., Fu J., Xing Y., Shen D., Ni Z. (2021). Triple and Two-Way
Reversible Shape Memory Polymer Networks with Body Temperature and
Water Responsiveness. Chem. Mater..

[47] Matsuda A., Sato J. I., Yasunaga H., Osada Y. (1994). Order-Disorder Transition
of a Hydrogel Containing an N-Alkyl Acrylate. Macromolecules.

[48] Feng X., Meitl M. A., Bowen A. M., Huang Y., Nuzzo R. G., Rogers J. A. (2007). Competing Fracture
in Kinetically Controlled Transfer
Printing. Langmuir.

[49] Linghu C., Mu T., Zhao W., Liu Y., Hsia K. J., Leng J., Gao H. (2025). Advancing Smart Dry
Adhesives with Shape Memory Polymers. Int. J.
Smart Nano Mater..

