# Supplementary material for: A Sharp Phase Transition Polymer for High Spatial Selectivity in Microtransfer Printing
Source: ACS Appl Polym Mater. 2025 Nov 19;7(24):16519–28. doi: 10.1021/acsapm.5c02952 (PMC12750521; doi:10.1021/acsapm.5c02952)
Supplement: Supplementary file 1 [file ap5c02952_si_001.pdf]

Supporting Information

A Sharp Phase Transition Polymer for High Spatial Selectivity Micro-Transfer Printing

Jingyang Zhang[1], Lizhou Yang[1], Qinhua Guo[1], Chenchen Zhang[1], Dong Lu[1,2], Yunda Wang[1,3*]*

[1] Smart Manufacturing Thrust, The Hong Kong University of Science and Technology (Guangzhou); Guangzhou, 511400, China.
[2] Lab of Future Technology, The Hong Kong University of Science and Technology (Guangzhou); Guangzhou, 511400, China.
[3] Department of Mechanical and Aerospace Engineering, The Hong Kong University of Science and Technology; Hong Kong SAR, 999077, China.

[*]Corresponding author. Email: ydwang@ust.hk; donglu@hkust-gz.edu.cn

Materials and Methods

Fourier Transform Infrared Spectroscopy (FTIR) Characterization

The infrared spectroscopic scans of SA and UDA mixtures, as well as the prepolymer solution samples with different curing times (5 min, 15 min, and 30 min), are carried out using a Bruker-Vertex 70V. During the scanning process, the number of scans is set to 16, while the data are recorded in the range from 2000 cm^{-1} to 500 cm^{-1}, covering the main infrared absorption peaks of the materials.

Differential Scanning Calorimetry (DSC) Characterization

Before the DSC measurement, the samples were pre-annealed at 60 °C and then cooled down naturally to room temperature. This pre-annealing process was used to erase any thermal history and ensure a consistent starting point for all samples undergoing DSC analysis.

Dynamic Mechanical Analysis (DMA) Characterization

Mechanical properties were measured on a TA Instruments DMA 850. Dynamic temperature sweep tests were conducted at a temperature ramping rate of 2 °C/min and a frequency of 1 Hz from 25 to 60 °C using samples of 25 mm long, 5 mm wide and ~ 1 mm thick, loaded onto the DMA with a 10 mm gap between the film grips. Ttensile tests were also carried out on the SPTP samples. The stress-strain curves of the copolymers were obtained at 50 °C and 25 °C at a stretching rate of 3.33 mm/s.

In-Site X-Ray Diffractometer (XRD) Characterization

In-site XRD tests were performed on thin-film SA-UDA samples using a X-Ray diffractometer (Malvern PANalytical Empyrean 3.0) at a temperature range from 25 °C to 45 °C with a heating rate of 2 °C/min. The separation of the amorphous diffraction and the crystalline peak was processed using OriginPro 8.

Fabrication of the micro-objects array on the original substrate

Polyimide structures:

A commercially available free-standing polyimide (PI) film (15 μm thick) is laminated onto a PDMS (SYLGARD 184, Dow Corning, America) substrate. To enhance the surface wettability, the PI surface undergoes oxygen plasma treatment at 50 W for 60 s.

AZ5214E photoresist is then spin-coated onto the PI surface and subsequently patterned. The surface is sequentially sputtered with 30 nm of Cr and 100 nm of Al, followed by the removal of the photoresist using an acetone solution. Finally, the PI is etched in a reactive ion etching chamber at a radio-frequency bias power of 200 W for 20 min, using an O_2/Ar gas mixture (flow rates: 250/50 sccm, pressure: 278.3 mTorr) to obtain discrete PI structures on the PDMS substrate.

Silicon cubes:

A 70-μm-thick silicon substrate is first adhered to a silicone gel-film substrate. Next, AZ5214E photoresist is spin-coated onto the silicon surface and patterned. Finally, deep reactive ion etching (DRIE) is used to etch the silicon, resulting in individual silicon chiplets.

AZ5214E structures:

A layer of AZ5214E photoresist is spin-coated onto the plasma-treated silicone gel-film substrate at 3000 rpm for 30 s, and then baked and exposed to 365 nm UV light. After exposure, the substrate is developed to form the desired photoresist patterns on the silicone gel-film surface.

Supplementary Text

Table S1. Composition and functional roles of the raw materials

Components	Ratio (part on weight)	Function	Structural Formula
SA	80	Sharp phase transition	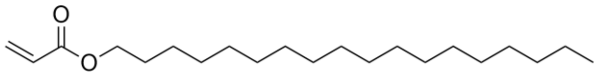
UDA	20	Framework	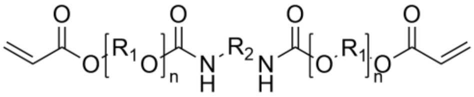
TMP-TA	1	Small molecular crosslinker	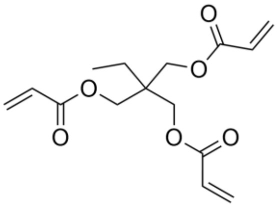
DMPA	1	Photoinitiator	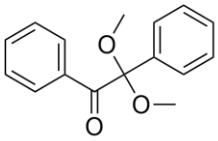
BP	0.5	Photoinitiator	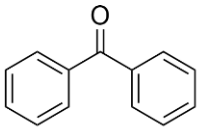

Table S1~~Table S1~~ shows the components, weight ratios, functions, and structural roles of the materials used in the SPTP formulation. Each component plays an important role in determining the material's properties and performance. The SPTP material consists of 80 parts SA, 20 parts UDA, 1 part trimethylolpropane triacrylate (TMP-TA), 1 part 2,2-dimethoxy-2-phenyl-acetophenone (DMPA), and 0.5 parts benzophenone (BP) by weight. SA has an optimal melting temperature of 32–34 °C and a distinct phase transition from crystalline to molten states, making it ideal for facilitating a clear transition from rigid to rubbery. The long-chain UDA serves as the polymer framework, which improves the elongation at break and enhances the toughness of the SPTP in its rubbery state. TMP-TA, as a trifunctional cross-linker, increases the stiffness of the resulting polymer under extensive strains. DMPA and BP, mixed in a 2:1 weight ratio,

act as co-photoinitiators to achieve complete curing throughout the bulk and on the surface of the films.

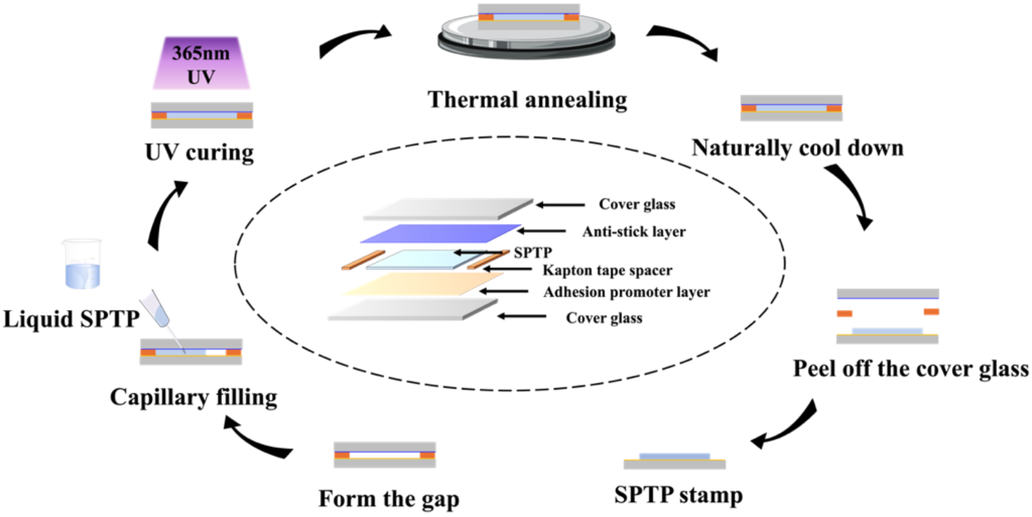

Figure S1. Molding method for fabricating the SPTP stamp.

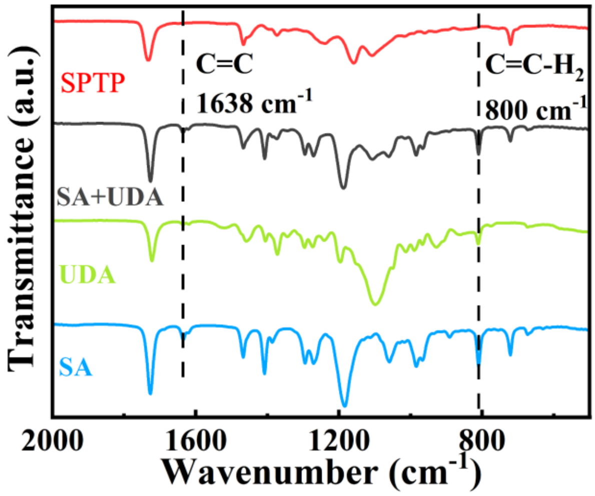

Figure S2. FTIR results confirming the successful synthesis of SPTP.

The peaks of 1638 cm^{-1} and 800 cm^{-1} carbon-carbon double bonds and carbonyl hydrogen disappeared, indicating the disappearance of the acrylic group, the completion of curing, and the successful synthesis of SPTP.

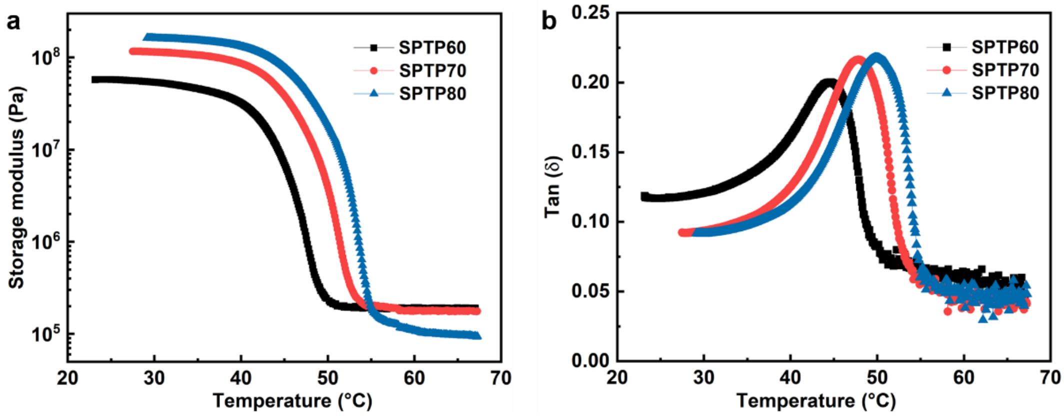

Figure S3. **a-b** Evolution of storage modulus and tan δ as a function of temperature, determined by DMA with temperature ramping from 22 to 68 °C at 2 °C min^{-1}.

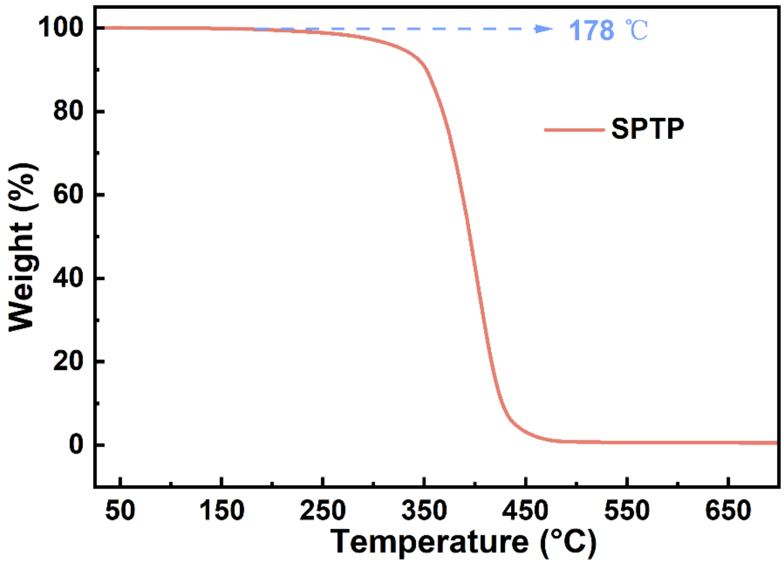

Figure S4. Thermogravimetric test of the SPTP material over a temperature range from 25 °C to 700 °C.

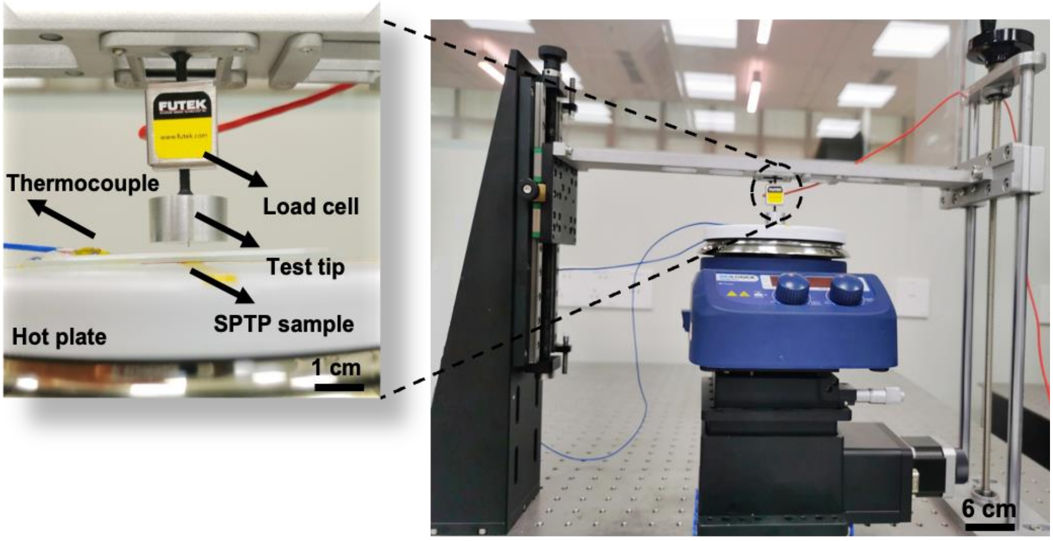

Figure S5. Customized test apparatus for measuring the adhesion force.

The setup includes an automatic Z-axis motion stage for precise interface contact and controlling the separation speed, a manual tip-tilt stage to control the angular position of the samples, enabling accurate alignment and positioning, a hot plate for heating, and a thermocouple for measuring the sample surface temperature.

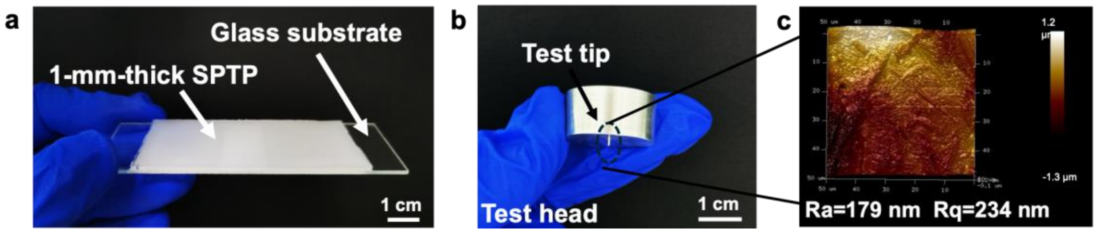

Figure S6. a Photograph of the customized SPTP stamp for adhesion force measurement. **b** Photograph of the test head. **c** The AFM image of the surface roughness of the test tip surface.

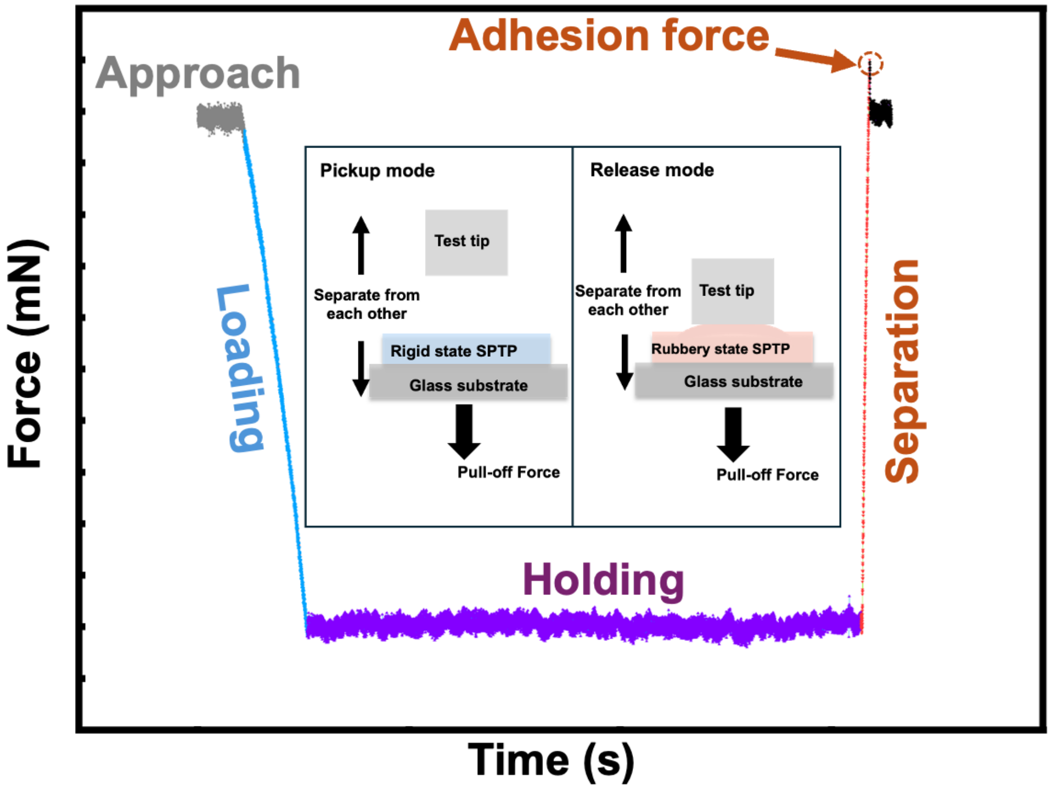

Figure S7. A representative force-time curve of the adhesion force tests. Inset: The details of pickup mode and release mode.

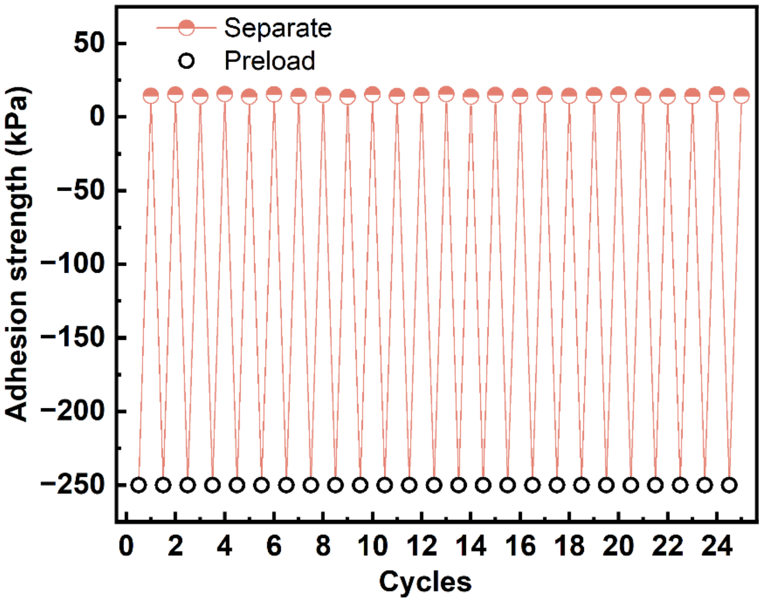

Figure S8. The adhesion performance over 25 cycles of adhesion and separation tests.

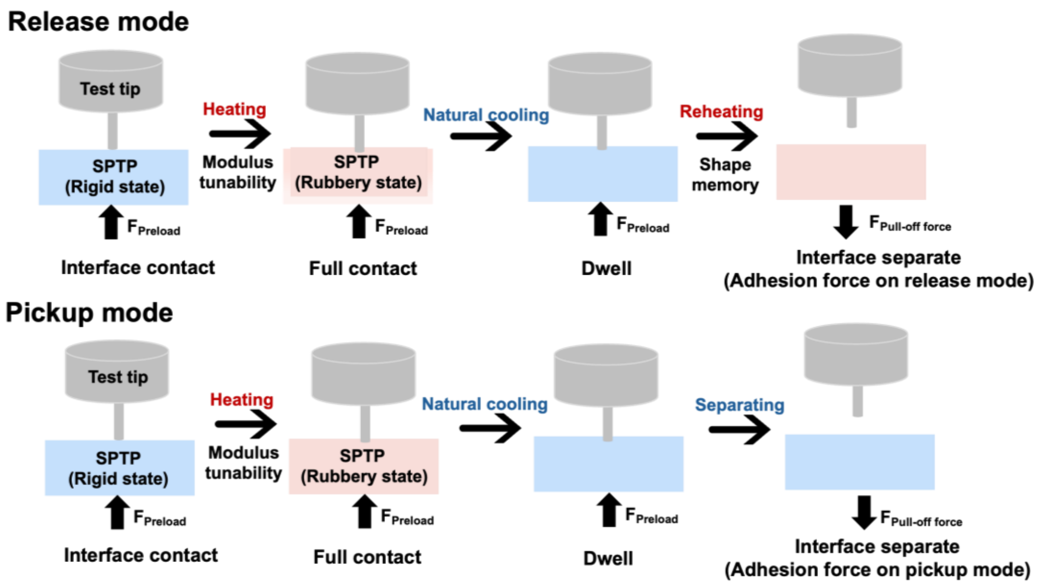

Figure S9. Illustrations of the adhesion test process for release mode and pickup mode adhesion of the SPTP stamp.

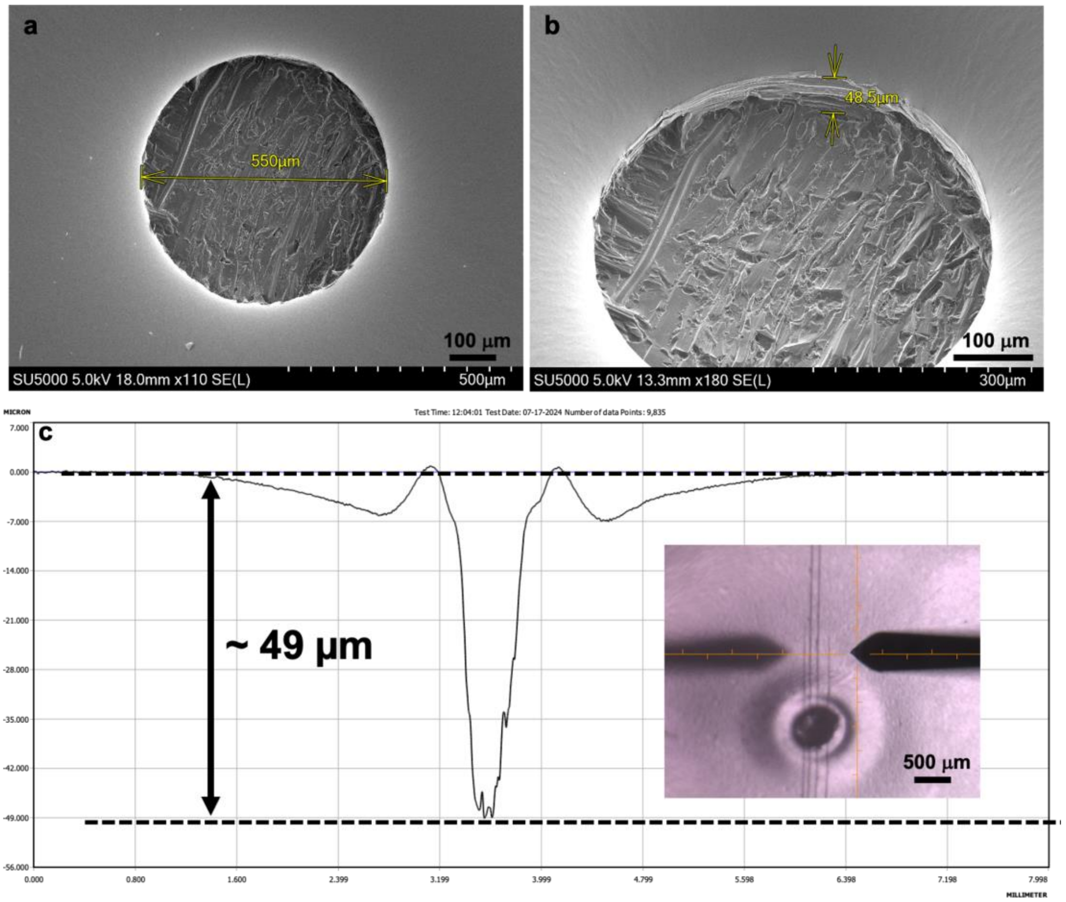

Figure S10. The depth of preload. SEM images of the SPTP stamp after 250 kPa preload, observed from different angles: **a** Vertical observation and **b** Oblique-angle observation. **c** The surface profile after the given preload. Inset: Microscopic images of surface morphologies after preload.

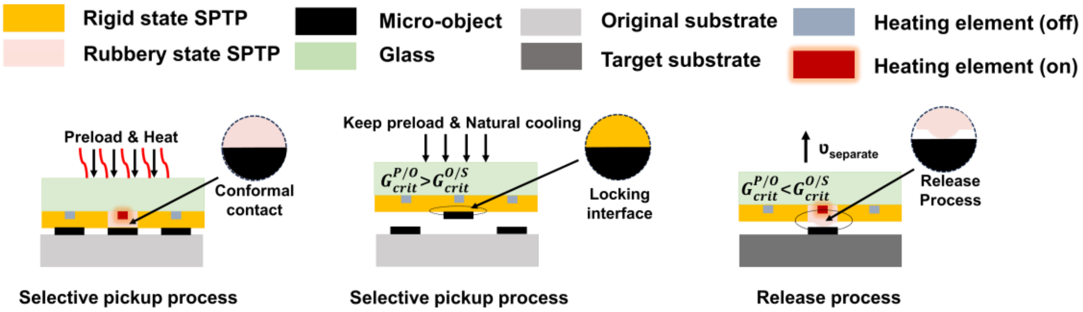

Figure S11. Schematic diagram of the selective pickup and release process. (The heating element is implemented using a microheater.)

Table S2. Comparison of the adhesion switchability of different flat surface stamps.

Materials	Max adhesion strength (Pa)	Adhesion switchability	Reference
SPTP	2802970	192	This work
PUA_G	1910.78	23	1
Epoxy SMP	113.9	83	2
PDMS-1	1530	12	3
PDMS-3	85	10	4
Epoxy SMP	3200	6	5
Ion gels	5000	4.9	6
Acrylate-based adhesive	1322000	117.5	7
Azobenzene-based	200000	50	8
GSMP	278000	29	9
Azobenzene poly(hexyl)acrylate	80000	12.75	10

References

1. Kim S, Liu N, Shestopalov AA. Contact printing of multilayered thin films with shape memory polymers. *ACS Nano* **16**, 6134-6144 (2022).
2. Huang Y*, et al.* Direct laser writing-based programmable transfer printing via bioinspired shape memory reversible adhesive. *ACS Applied Materials & Interfaces* **8**, 35628-35633 (2016).
3. Kim T-H*, et al.* Kinetically controlled, adhesiveless transfer printing using microstructured stamps. *Applied Physics Letters* **94**, 113502 (2009).
4. Carlson A*, et al.* Shear-enhanced adhesiveless transfer printing for use in deterministic materials assembly. *Applied Physics Letters* **98**, 264104 (2011).
5. Linghu C*, et al.* Universal smp gripper with massive and selective capabilities for multiscaled, arbitrarily shaped objects. *Science Advances* **6**, eaay5120 (2020).
6. Wang S*, et al.* A wearable glove with electrothermal-controlled ionogels for adhesive gripping. *Advanced Intelligent Systems* **6**, 2300127 (2024).
7. Guo C*, et al.* Large-scale programmable assembly of functional micro-components for advanced electronics via light-regulated adhesion and polymer growth. *npj Flexible Electronics* **6**, 44 (2022).
8. Lee T-H, Han G-Y, Yi M-B, Kim H-J, Lee J-H, Kim S. Rapid photoresponsive switchable pressure-sensitive adhesive containing azobenzene for the mini-light emitting diode transfer process. *ACS Applied Materials & Interfaces* **13**, 43364-43373 (2021).
9. Tan D*, et al.* Switchable adhesion of micropillar adhesive on rough surfaces. *Small* **15**, 1904248 (2019).
10. Zhou Y*, et al.* Light-switchable polymer adhesive based on photoinduced reversible solid-to-liquid transitions. *ACS Macro Letters* **8**, 968-972 (2019).
